# UHRF1 downmodulation enhances antitumor effects of histone deacetylase inhibitors in retinoblastoma by augmenting oxidative stress‐mediated apoptosis

**DOI:** 10.1002/1878-0261.12607

**Published:** 2019-12-13

**Authors:** Jong Kyong Kim, Guangyan Kan, Yu Mao, Zhixuan Wu, Xionghong Tan, Heng He, Chunsik Lee

**Affiliations:** ^1^ State Key Laboratory of Ophthalmology Zhongshan Ophthalmic Center Sun Yat‐sen University Guangzhou China

**Keywords:** chemotherapy, drug sensitivity, HDAC inhibitors, retinoblastoma, UHRF1

## Abstract

Identification of new genetic pathways or molecular targets that sensitize cancer cells to chemotherapeutic drugs may improve the efficacy of current chemotherapy. Here, we report that downmodulation of UHRF1 (ubiquitin‐like with PHD and RING finger domains 1) in retinoblastoma (RB) cells increases the sensitivity to histone deacetylase (HDAC) inhibitors, augmenting apoptotic cell death. We found that UHRF1 depletion downregulates two redox‐responsive genes GSTA4 (glutathione *S*‐transferase α4) and TXN2 (thioredoxin‐2) in RB cells, and increases the basal level of intracellular oxidative stress. Antioxidant treatment significantly reduced both basal and HDAC inhibitor‐induced DNA damage and apoptosis in UHRF1‐depleted cells. Knockdown of GSTA4 or TXN2 sensitized RB cells to HDAC inhibitors, demonstrating that GSTA4 and TXN2 play key roles in redox homeostasis in RB cells and the susceptibility to HDAC inhibitor treatment upon UHRF1 depletion. In human primary RB, GSTA4 and TXN2 proteins were found to be mostly elevated along with high UHRF1 expression. In addition to augmentation of apoptosis in UHRF1‐depleted RB cells, we also show that UHRF1 downmodulation derepresses the expression of photoreceptor‐specific genes in RB cells in cooperation with a HDAC inhibitor MS‐275 and promotes neuron‐like differentiation. However, further investigation revealed that the enhanced growth‐inhibitory effects of MS‐275 in UHRF1‐depleted cells were still mainly due to robust apoptosis induction rather than differentiation‐mediated growth arrest. Consistent with our findings, UHRF1 depletion in RB cells increased the therapeutic efficacy of MS‐275 in murine orthotopic xenografts. These results provide a novel basis for potential benefits of UHRF1 targeting for RB treatment.

## Graphical abstract

This study presents two novel tumor‐promoting functions of UHRF1 in retinoblastoma (RB). UHRF1 regulates reactive oxygen species (ROS)‐detoxifying genes to counteract the accumulation of intracellular ROS and thereby protects the cells from histone deacetylase (HDAC) inhibitor‐induced apoptosis. On the other hand, UHRF1 participates in repression of photoreceptor differentiation as a corepressor in a multiprotein complex containing HDAC to repress photoreceptor‐specific genes in RB cells.

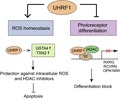

**Abbreviations**
GSTA4: glutathione *S*‐transferase α4
HDAC: histone deacetylase
NaBu: sodium butyrate
NAC: *N*‐acetylcysteine
PDL: poly‐d‐lysine
RA: all‐trans retinoic acid
RB: retinoblastoma
RCVRN: recoverin
ROS: reactive oxygen species
RXRG: retinoid X receptor γ
SAHA: suberoylanilide hydroxamic acid
TXN2: thioredoxin‐2
UHRF1: ubiquitin‐like with PHD and RING finger domains 1

## 1 Introduction

Retinoblastoma (RB) is a major intraocular cancer occurring in children and is initiated by inactivation of the *RB1* tumor suppressor gene in the developing retina (Dimaras and Corson, 2019). As a standard treatment option, chemotherapy has been widely used in combination with various types of adjuvant focal therapy to save the eye and reduce the long‐term risks of developing secondary tumors (Chan *et al.*, 2005; Wyse *et al.*, 2016). However, the current chemotherapy for RB has limitations such as drug resistance and adverse side effects due to toxicity of the drugs as the common chemotherapy regimens include several genotoxic drugs such as DNA topoisomerase II inhibitors and DNA crosslinking agents (Gombos *et al.*, 2007; Mulvihill *et al.*, 2003). Therefore, other small molecule inhibitors have been searched and tested for RB treatment in preclinical studies (Pritchard *et al.*, 2016). One class of such inhibitors is histone deacetylase (HDAC) inhibitors, which have been under continued development for treating many diseases including cancers and neurological disorders since their excellent therapeutic efficacy was proven for treating cutaneous T cell lymphoma (Duvic *et al.*, 2007; Falkenberg and Johnstone, 2014; Zagni *et al.*, 2017). HDAC inhibitors are known to exert diverse anticancer effects including apoptosis, cell cycle arrest, and differentiation although the detailed mechanisms of action may be highly varied depending on the types of the inhibitors and cancer cells tested (Falkenberg and Johnstone, 2014). As cancer cells with elevated E2F1 activity have been shown to be sensitive to HDAC inhibitor‐induced cell death (Zhao *et al.*, 2005) and *RB1* inactivation in RB results in deregulated E2F1 activity, several studies have investigated the effects of HDAC inhibitors on RB cell death (Dalgard *et al.*, 2008; Karasawa and Okisaka, 2004; Poulaki *et al.*, 2009). Butyrate and trichostatin A induced morphological changes and apoptosis of Y79 RB cells preceded by the increase in histone H3 acetylation (Karasawa and Okisaka, 2004). Similar growth‐inhibitory effects were observed by the treatment of suberoylanilide hydroxamic acid (SAHA) and MS‐275 in several RB cell lines, and MS‐275 was found to be efficacious for reducing tumor burden in two preclinical animal models of RB (Dalgard *et al.*, 2008).

Considering the importance of chemotherapy for RB treatment and continuous effort for development of new drugs, identification of novel genetic pathways or molecular targets that may sensitize RB cells to currently available chemotherapeutic drugs may provide an alternative strategy to improve the efficacy and applicability of the chemotherapy. Previously, we demonstrated that downmodulation of UHRF1 (ubiquitin‐like with PHD and RING finger domains 1) in RB cells enhances the sensitivity to standard chemotherapeutic drugs such as etoposide by impairing DNA repair and consequently resulting in more robust apoptotic cell death (He *et al.*, 2018). The UHRF1 is highly expressed in RB without detectable expression in normal retina and has been proposed to mediate epigenetic deregulation of the genes critical for RB tumorigenesis (Benavente *et al.*, 2014). Although our comprehensive DNA methylome analyses revealed that the tumor‐promoting functions of UHRF1 in RB are largely independent of its role in DNA methylation (Kan *et al.*, 2017), UHRF1 may exert other functions as an epigenetic regulator in RB as it is known to interact with modified/unmodified histones and several chromatin modifiers to regulate gene expression (Bronner *et al.*, 2013; Unoki, 2011). In fact, a previous study reported that UHRF1 forms a complex with HDAC1 and binds to methylated promoters of tumor suppressor genes such as *CDKN2A*, suggesting that UHRF1 mediates the tumor suppressor repression in cooperation with HDAC1 (Unoki *et al.*, 2004). Investigating the roles for UHRF1 in RB cells in response to HDAC inhibitors may provide a novel insight into the eligibility of UHRF1 as a potential target whose downmodulation can sensitize the cells to HDAC inhibitors as is the case with conventional chemotherapeutic drugs. Therefore, we examined the effects of UHRF1 depletion on the sensitivity to HDAC inhibitors in RB cells and the molecular mechanisms underlying the changes in drug sensitivity. Furthermore, we evaluated the therapeutic efficacy of MS‐275 treatment upon UHRF1 downmodulation in a murine orthotopic xenograft model of RB.

## 2 Materials and methods

### 2.1 Cell culture and reagents

Y79 and Weri‐Rb1 were obtained from American Type Culture Collection (ATCC, Manassas, VA, USA), and SO‐Rb50 was established at the Zhongshan Ophthalmic Center (ZOC) (Yi and Jie, 1990). All RB cell lines were maintained in RPMI‐1640 containing 10% FBS and penicillin‐streptomycin (Gibco, Waltham, MA, USA). SAHA and MS‐275 were purchased from Selleck and dissolved in dimethyl sulfoxide (DMSO) at a concentration of 10 mm. Sodium butyrate (NaBu; Selleck, Houston, TX, USA) was prepared in sterile water (200 mm), and all‐trans retinoic acid (RA; Selleck) was dissolved in DMSO (200 mm). The oxidative stress indicator CM‐H2DCFDA (Invitrogen, Waltham, MA, USA) was reconstituted as a 2 mm stock in DMSO, and *N*‐acetylcysteine (NAC; Sigma, St. Louis, MO, USA) was dissolved in sterile water (500 mm). Cells were treated with drugs to final concentrations in culture media with vehicle controls set up in parallel. Stable (long‐term) UHRF1‐knockdown cells were used for most studies unless indicated otherwise, and freshly generated at each time of experiments by lentiviral shRNA transduction mostly using the shUHRF1‐1 clone and subsequent selection on puromycin for 7–9 days beyond the short‐term knockdown (4 days post‐lentiviral infection) as described previously (Kan *et al.*, 2017). Lentiviral knockdown in this study was performed using the following shRNA clones from Dharmacon (Lafayette, CO, USA):

shUHRF1‐1: AAGAAGGAACGAATCAAAGGC
shUHRF1‐2: AAAGCAGTTGAGAGCCAGCGC
shGSTA4 #654: ATAAGGAGAGCAGAAAGACGC
shGSTA4 #839: TATGGCCTAAAGATGTTGTAG
shTXN2 #200: TTAAAGGTTGTCAAGGAGATC
shTXN2 #202: AATATCCACCTTGGCCATCAC
shRXRG: TAGTTCATGTTTCCAATCCCG

### 2.2 Human retinoblastoma tissues

Human primary RB tissues were obtained from the ocular tumor division and department of pathology at the ZOC. The study with human clinical samples conformed to the standards set by the Declaration of Helsinki and was approved by the ZOC institutional review board. All human specimens used for this study were de‐identified, and written informed consent forms were obtained.

### 2.3 Cell viability and apoptosis assays

Y79 cells were plated at a density of 5 × 10^6^ cells/dish in duplicate per treatment group at each experiment. After cells were treated with drugs at indicated concentrations, cell viability was determined by direct live cell counting based on trypan blue exclusion. For apoptosis assays, cells were harvested by taking all suspension cells in the medium and apoptotic cell populations were detected by flow cytometry using the Annexin V‐FITC Apoptosis Kit (Roche, Basel, Switzerland) according to the manufacturer's instruction.

### 2.4 Western blot

Cleared lysates (25–30 µg) were subjected to 12.5% SDS/PAGE. Antibodies for western blots are as follows: UHRF1 (sc‐166898; Santa Cruz, Santa Cruz, CA, USA); γH2AX (9718), cleaved PARP (9541), TXN (2429), TXN2 (14907), p38 (8690), phospho‐p38 (Thr180/Tyr182) (4511), acetyl‐histone H3 (Lys9) (9649), total histone H3 (9715), HDAC1 (34589), and HDAC2 (57156) from Cell Signaling Technology (Danvers, MA, USA); acetyl‐histone H3 (06‐599) and acetyl‐histone H4 (06‐866) from Millipore (Burlington, MA, USA); actin (A1978; Sigma), GSTA4 (ab134919; abcam, Cambridge, MA, USA), and caspase‐3 (40924; Active Motif, Carlsbad, CA, USA).

### 2.5 Intracellular reactive oxygen species detection

Cells were plated in poly‐d‐lysine (PDL)‐coated vessels and incubated with 10 µm CM‐H2DCFDA (redox‐sensitive probe) in complete media at 37 °C for 30 min in dark, and then washed with PBS twice to remove free probe. Reactive oxygen species (ROS) fluorescence was detected by fluorescence microscopy (Leica DMi8, Wetzlar, Germany).

### 2.6 Differentiation assay

The differentiation potential of the stable Y79 control and UHRF1‐knockdown cells was examined by plating the cells on PDL‐coated flasks in the regular culture medium for 24 h, followed by switching to serum‐free neurobasal medium containing G‐5 supplement (Gibco) with or without 1 µm MS‐275 for an additional 24 h.

### 2.7 ChIP and quantitative RT‐PCR

ChIP was performed by using SimpleChIP Enzymatic Chromatin IP kit (Cell Signaling Technology) according to the manufacturer's instructions. The antibodies used for ChIP are as follows: UHRF1 (612264; BD Biosciences, Franklin Lakes, NJ, USA); total histone H3 (4620), HDAC1 (34589), and HDAC2 (57156) from Cell Signaling Technology; acetyl‐histone H3 (06‐599) and acetyl‐histone H4 (06‐866) from Millipore. ChIP DNA was analyzed by PCR along with input DNA and quantified as % input for each target before calculation of fold changes or ratios of the signals among different experimental groups. The qRT‐PCR was performed by analyzing samples in triplicate using at least three independent sets of cDNA. The results were normalized by the expression level of actin as an internal control. The primer sequences used for the qRT‐PCR and ChIP assays are listed in supplementary data (Table S1).

### 2.8 RNA‐sequencing analysis

Total RNA was isolated from stable Y79 shCTL and shUHRF1 cells using RNeasy Plus Mini kit (Qiagen, Germantown, MD, USA). RNA‐seq libraries were prepared using the standard Illumina protocols and subjected to 50 bp single‐end sequencing on an Illumina HiSeq 2500 platform by BerryGenomics (Beijing, China). The differentially expressed genes between control and UHRF1‐knockdown cells were detected by edgeR package, based on the analysis criteria of ≥ twofold change in expression and a FDR (false discovery rate) threshold of 0.05. For the heat map analysis for a subgroup of the differentially expressed genes, relevant genes represented by at least > 1 FPKM (fragments per kilobase per million mapped fragments) in shUHRF1 cells were selected from the total differentially expressed genes, based on the Gene Ontology (GO) annotations and partial literature search. The GO analysis for differentially expressed genes was performed by topGO package using the GO database downloaded from geneontology.org with a *P*‐value < 0.05. For the gene set enrichment analysis, r package clusterProfiler was used with gene sets downloaded from Molecular Signatures Database (MSigDB). Enrichment scores were calculated based on the log2 fold change in shUHRF1 cells over control groups by walking down the whole transcriptomic profile with an increasing running‐sum statistic when a gene is present in the gene set while decreasing the statistic when the gene is not in that gene set. The RNA‐seq data in this study were deposited in the NCBI Gene Expression Omnibus (GEO) database under the accession number http://www.ncbi.nlm.nih.gov/geo/query/acc.cgi?acc=GSE135424.

### 2.9 Therapeutic study on orthotopic xenografts

Orthotopic xenografts of RB were established by injecting control or UHRF1‐knockdown Y79 cells (2 × 10^5^ in 2 µL volume) into the vitreous of the right eye while leaving the left eye uninjected as a control, using 6‐ to 7‐week‐old BALB/c female nude mice (Model Animal Research Center, Nanjing University). The intravitreal transplantation was performed for 12 mice per round to ensure the quality of the injected cells by following the procedure described previously (Tschulakow *et al.*, 2016). On day 13 post‐transplantation, tumor formation was examined by *in vivo* retinal imaging with a Micron IV retinal microscope (Phoenix Research Lab, Pleasanton, CA, USA) after sedation of animals. Only the mice with detectable tumors were subjected to the treatment with MS‐275 (10 mg·kg^−1^) by intraperitoneal injection every other day for 2 weeks after grouping the mice with a similar tumor burden between control and UHRF1‐knockdown xenografts based on the retinal imaging results. The next day after the 2 weeks' treatment, tumor‐burdened eyes were analyzed for the average tumor area per eye by modifying the procedure described previously (Dalgard *et al.*, 2008). Briefly, nine to ten representative sections spanning the whole eye globe were used for hematoxylin and eosin (H&E) staining per eye by taking every 50th section (4‐µm‐thick each) from one end of the eyeball to the other end. The image of each section was used for tumor area quantification in pixels with imagej software (NIH, Bethesda, MD, USA), and tumor burden per mouse was calculated by taking the average tumor area per section from the nine to ten sections representing different levels of the whole eyeball. Some of the serial sections were subjected to immunostaining with anti‐UHRF1 antibody (sc‐373750; Santa Cruz), following the procedure described previously (Kan *et al.*, 2017). All animal studies were conducted with the approval of the Sun Yat‐sen University Institutional Animal Care and Use Committee.

### 2.10 Statistical analyses

Statistical significance was determined from at least three independent experiments by two‐tailed unpaired student's *t*‐test using graphpad prism (GraphPad Software, La Jolla, CA, USA) unless indicated otherwise in the legend.

## 3 Results

### 3.1 UHRF1 depletion sensitizes retinoblastoma cells to HDAC inhibitors

**Figure 1:**
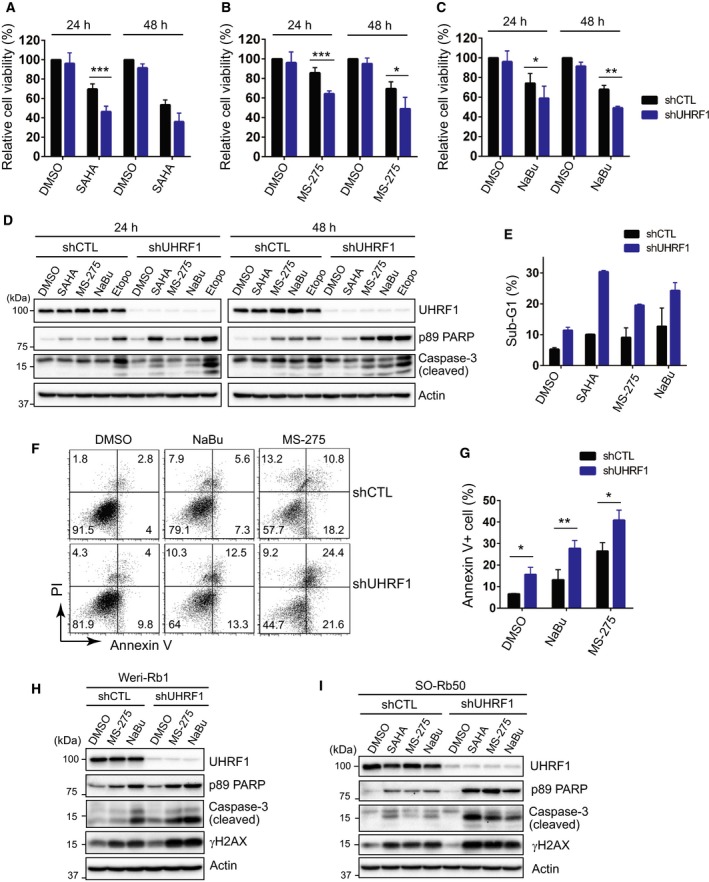
UHRF1 depletion sensitizes RB cells to HDAC inhibitors. (A–C) Relative cell viability determined by live cell counting after treatment with HDAC inhibitors. Stable control‐knockdown (shCTL) and UHRF1‐knockdown (shUHRF1) Y79 cells were treated with 1 µm SAHA (A), 1 µm MS‐275 (B), and 1 mm NaBu (C) for the indicated time. The results are shown as the mean ± standard deviation (SD) of % fold changes from three independent experiments, relative to the cell viability in shCTL cells treated with vehicle (DMSO). *P < 0.05, **P < 0.01, ***P < 0.001: unpaired Student's *t*‐test (two‐tailed). (D) Immunoblots for indicated proteins in Y79 shCTL and shUHRF1 cells after exposure to 1 µm SAHA, 1 µm MS‐275, and 1 mm NaBu for the indicated time. Cells treated with 10 µm etoposide (Etopo) for 24 h are shown as common positive controls for apoptosis in parallel. (E) Percentages of sub‐G1 population determined by flow cytometry in shCTL and shUHRF1 Y79 cells treated with HDAC inhibitors as in (D) for 24 h. The results are shown as the mean ± SD from triplicate experiments. (F) Annexin V^+^ apoptotic cell populations detected by flow cytometry after treatment with 1 mm NaBu or 1 µm MS‐275 for 48 h. The percentage of population in each quadrant is shown. (G) Percentages of annexin V^+^ cells determined in (F). The results are shown as the mean ± SD from three independent experiments. *P < 0.05, **P < 0.01: unpaired Student's *t*‐test (two‐tailed). (H) Immunoblots in Weri‐Rb1 shCTL and shUHRF1 cells treated with 1 µm MS‐275 or 1 mm NaBu for 20 h. (I) Immunoblots in SO‐Rb50 shCTL and shUHRF1 cells after exposure to 1 µm SAHA, 1 µm MS‐275, and 1 mm NaBu for 28 h.

We examined the relative viability of control and UHRF1‐knockdown Y79 RB cells in response to several HDAC inhibitors (Fig. 1A–C). UHRF1‐depleted cells showed a consistent increase in sensitivity to all tested HDAC inhibitors. As HDAC inhibitors can affect cell viability and proliferation by several different mechanisms (Falkenberg and Johnstone, 2014), we first investigated whether enhanced apoptosis contributes to the higher sensitivity observed in UHRF1‐depleted Y79 cells. HDAC inhibitors were found to induce higher apoptotic responses in the UHRF1‐knowndown cells as demonstrated by elevated levels of cleaved caspase‐3 and poly(ADP‐ribose) polymerase (PARP) (Fig. 1D). The increased sub‐G1 and annexin V^+^ apoptotic populations in UHRF1‐knockdown cells further supported the finding that UHRF1 depletion renders RB cells sensitized to HDAC inhibitors (Fig. 1E–G). The higher sensitivity to HDAC inhibitors following UHRF1 depletion was also observed in other RB cells such as Weri‐Rb1 and SO‐Rb50 (Fig. 1H,I), and could be detected by acute UHRF1 knockdown as well in Y79 and Weri‐Rb1 cells (Fig. S1A,B). When we examined the sensitization to HDAC inhibitors using another shRNA clone targeting UHRF1 (shUHRF1‐2) in Y79 cells, we observed higher basal apoptosis than shUHRF1‐1 clone, which appeared to make the HDAC inhibitor sensitivity with shUHRF1‐2 clone much less clear (Fig. S1C). However, we have consistently detected the modest increase in DNA damage signal and cleaved PARP in response to HDAC inhibitors upon shUHRF1‐2 clone‐mediated knockdown at each time of experiments. This observation raises a possibility that the discrepancy in basal apoptosis between the two shRNA clones may reflect certain levels of off‐target effects. As the reduced cell viability determined by live cell counts relative to control may be attributed to cell cycle arrest as well, we have examined cell cycle profiles for both control and UHRF1‐knockdown cells upon HDAC inhibitor treatment; however, we have not observed any clear cell cycle arrest in UHRF1‐depleted cells in the presence and absence of HDAC inhibitors (Fig. S2).

### 3.2 UHRF1 depletion deregulates redox‐responsive genes in retinoblastoma cells

**Figure 2:**
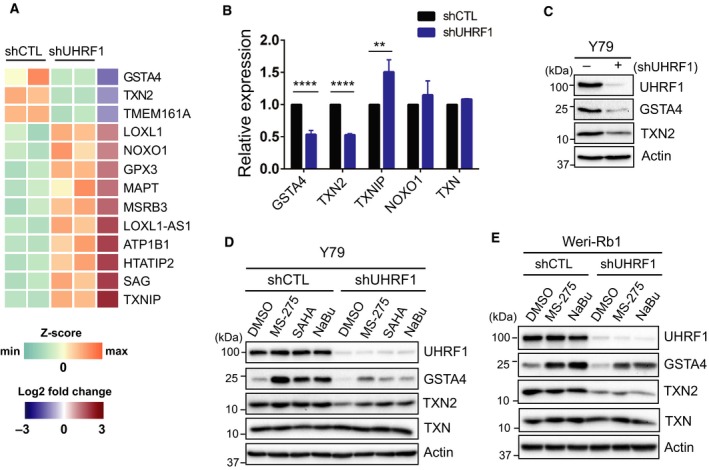
UHRF1 depletion deregulates redox‐responsive genes in RB cells. (A) Heat map of differentially expressed genes related to redox and oxidative stress in shUHRF1 Y79 cells. Each column represents an independent replicate. (B) qRT‐PCR analysis for the relative expression of indicated genes in Y79 shCTL and shUHRF1 cells. The bar graph is shown as the mean ± SD of fold changes from four independent experiments, relative to the normalized level in shCTL cells. **P < 0.01, ****P < 0.0001: unpaired Student's *t*‐test (two‐tailed). (C) Basal expression changes in GSTA4 and TXN2 upon UHRF1 knockdown in Y79 cells. (D) Immunoblots for indicated proteins in Y79 shCTL and shUHRF1 cells after exposure to 1 µm MS‐275, 1 µm SAHA, and 1 mm NaBu for 24 h. (E) Immunoblots in Weri‐Rb1 shCTL and shUHRF1 cells treated with 1 µm MS‐275 or 1 mm NaBu for 20 h.

To understand how UHRF1 depletion enhances apoptosis in RB cells in response to HDAC inhibitors, we performed RNA‐sequencing to identify differentially expressed genes upon stable UHRF1 knockdown in Y79 cells. The gene expression analysis allowed us to identify total 829 differentially expressed genes in UHRF1‐knockdown cells (Table S2). As ROS accumulation is implicated in HDAC inhibitor‐induced apoptosis in cancer cells (Rosato *et al.*, 2003; Ungerstedt *et al.*, 2005), we sorted redox‐related genes by GO annotations from the total 829 differentially expressed genes and found the marked expression changes in several redox‐responsive genes (Fig. 2A and Table S2). Although a recent study reported that TXNIP (thioredoxin interacting protein) is epigenetically repressed by UHRF1 and has a tumor suppressor role in renal cell carcinoma cells (Jiao *et al.*, 2019), our further validation in RB cells revealed that basal TXNIP expression and biological responses to HDAC inhibitors are inconsistent in Y79 and Weri‐Rb1 cells upon UHRF1 depletion (data not shown), suggesting that the role of TXNIP may be tumor‐specific. However, expression of GSTA4 (glutathione *S*‐transferase α4) and TXN2 (thioredoxin‐2) was consistently low at both transcript and protein levels in UHRF1‐depleted cells (Fig. 2B,C). When we examined the expression changes for these genes in response to HDAC inhibitors in UHRF1‐depleted RB cells, the basal and HDAC inhibitor‐induced levels of GSTA4 were substantially lower in the UHRF1‐knockdown cells than in control cells (Fig. 2D,E). Furthermore, the mitochondrial thioredoxin TXN2 was found to be reduced in UHRF1‐knockdown cells, while cytosolic TXN remained constant compared to the control counterparts (Fig. 2D,E). The decreased GSTA4 and TXN2 protein was also observed upon acute UHRF1 knockdown and could be detected with another shUHRF1 clone‐mediated knockdown (Fig. S3A–C). Of note, our RNA‐sequencing results did not reveal any significant expression changes in genes involved in intracellular ROS generation, pointing to a possibility that UHRF1 depletion mainly deregulates ROS‐detoxifying genes represented by GSTA4 and TXN2.

### 3.3 Downregulation of GSTA4 and TXN2 by UHRF1 depletion contributes to enhanced sensitivity to HDAC inhibitors in retinoblastoma cells

**Figure 3:**
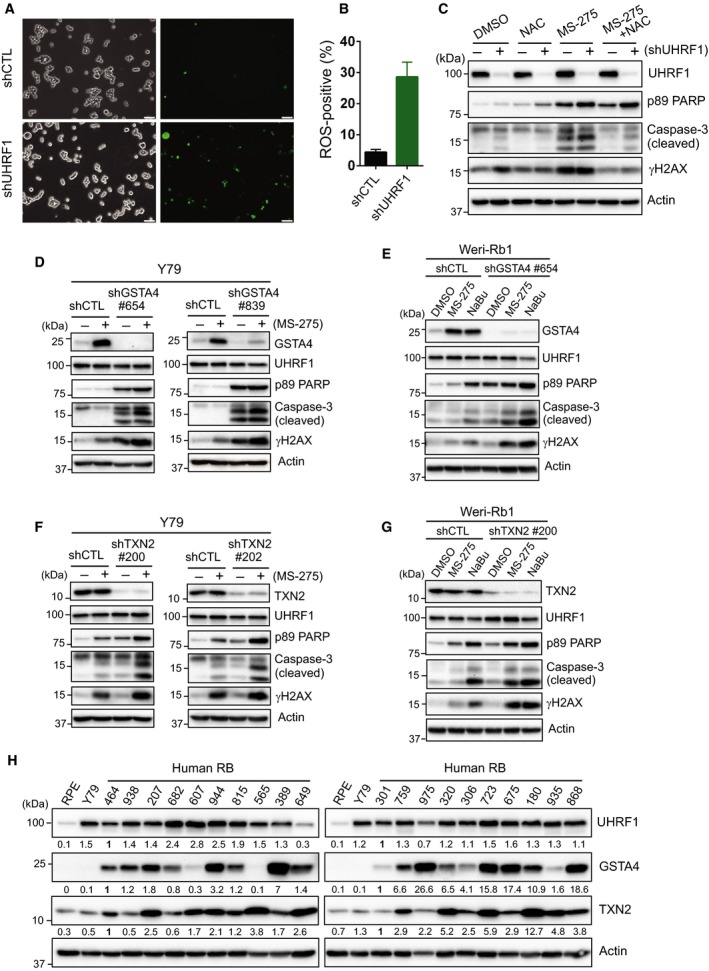
Downregulation of GSTA4 and TXN2 by UHRF1 depletion contributes to enhanced sensitivity to HDAC inhibitors in RB cells. (A) Detection of basal intracellular ROS levels by reactivity with a fluorescent probe in Y79 shCTL and shUHRF1 cells. ROS (green fluorescence)‐positive cells are shown along with phase contrast images of the cells on the left. Scale bars: 50 µm. (B) Quantification of ROS‐positive cells shown in (A). Over 900 total cells from five randomly selected fields were evaluated for ROS positivity. The data represent the mean ± SD from two replicates. (C) Immunoblots for indicated proteins in Y79 control (−) and UHRF1‐knockdown (+) cells subjected to a single or combined treatment with 10 mm NAC and 1 µm MS‐275 for 48 h. (D) Immunoblots in Y79 shGSTA4 (clones #654 and #839) cells treated with 1 µm MS‐275 for 24 h. (E) Immunoblots in Weri‐Rb1 shGSTA4 cells treated with 1 µm MS‐275 or 1 mm NaBu for 20 h. (F) Immunoblots in Y79 shTXN2 (clones #200 and #202) cells treated with 1 µm MS‐275 for 48 h. (G) Immunoblots in Weri‐Rb1 shTXN2 cells treated as in (E). (H) Expression of GSTA4 and TXN2 in human RB tumor lysates. Relative abundance of proteins determined by densitometry is shown below each panel of the blots, in comparison with the first human RB sample on each panel. RPE: retinal pigment epithelium.

The downregulation of GSTA4 and TXN2 upon UHRF1 depletion suggested that these cells may encounter increased oxidative stress due to the impaired ROS detoxification, contributing to enhanced susceptibility to ROS‐mediated apoptosis driven by HDAC inhibitors. In line with the possibility, a higher level of intracellular ROS was detected in the UHRF1‐depleted cells (Fig. 3A,B), and treatment with an antioxidant NAC reduced both basal and MS‐275‐induced DNA damage signal and caspase‐3 activation in UHRF1‐knockdown cells, while PARP cleavage was not reduced by NAC treatment (Fig. 3C). Consistent with the increased oxidative stress in UHRF1‐depleted cells, p38 phosphorylation was significantly higher in HDAC inhibitor‐treated UHRF1‐knockdown cells, while there were no remarkable changes in expression of NRF2, a regulator of antioxidant defense, in the presence and absence of HDAC inhibitors in UHRF1‐depleted cells (Fig. S4A–C). Knockdown of GSTA4 alone in RB cells markedly increased the DNA damage and apoptotic signals even without treatments, and HDAC inhibitors further augmented the apoptosis (Fig. 3D,E). Similarly, TXN2 depletion sensitized RB cells to HDAC inhibitors (Fig. 3F,G), indicating that impaired ROS detoxification in UHRF1‐knockdown cells may account for the higher sensitivity to HDAC inhibitors. In human primary RB tissues, GSTA4 and TXN2 proteins were found to be mostly elevated along with high UHRF1 expression (Fig. 3H), supporting a role for UHRF1 in redox homeostasis mediated by GSTA4 and TXN2 although biological responses including gene expression changes in the UHRF1‐knockdown cells may not fully reflect what occurs in tumors.

### 3.4 UHRF1 depletion derepresses expression of photoreceptor genes in retinoblastoma cells

**Figure 4:**
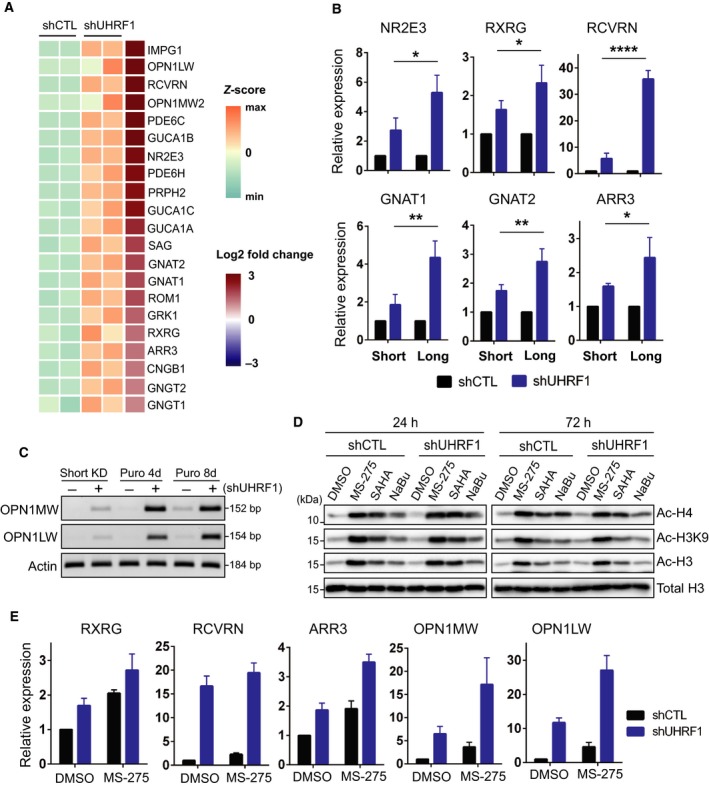
UHRF1 depletion derepresses expression of photoreceptor genes in RB cells. (A) Heat map of differentially expressed genes related to photoreceptor and phototransduction in shUHRF1 Y79 cells. (B) Relative expression of photoreceptor genes in control and UHRF1‐knockdown Y79 cells subjected to short‐term knockdown (Short; 4 days post‐lentiviral infection without selection on puromycin) or long‐term knockdown (Long; 8 days' selection on puromycin in addition to the initial 4 days postinfection). The qRT‐PCR data are shown as the mean ± SD of fold changes from four independent experiments, relative to the normalized level in each control‐knockdown group. *P < 0.05, **P < 0.01, ****P < 0.0001: unpaired Student's *t*‐test (two‐tailed). (C) RT‐PCR analysis for indicated genes in control and UHRF1‐knockdown Y79 cells along the progression of selection on puromycin (puro) from short‐term knockdown (KD). (D) Immunoblots for indicated histones in Y79 shCTL and shUHRF1 cells after exposure to 0.5 µm MS‐275, 1 µm SAHA, and 1 mm NaBu for the indicated time. (E) Relative expression of photoreceptor genes in shCTL and shUHRF1 Y79 cells (puro 8d) treated with 0.5 µm MS‐275 for 48 h. The qRT‐PCR data are shown as the mean ± SD of fold changes from three independent experiments, relative to the normalized level in DMSO‐treated shCTL cells.

UHRF1 is known to be a transcriptional corepressor that silences genes involved in cellular differentiation (Enane *et al.*, 2018). Interestingly, our RNA‐sequencing analysis revealed that many photoreceptor‐specific genes were upregulated in UHRF1‐depleted cells (Fig. 4A and Fig. S5, Table S2), which is consistent with previous reports that human RB displays photoreceptor‐like features (McEvoy *et al.*, 2011; Xu *et al.*, 2009) and Y79 cells differentiate predominantly into a neuronal, photoreceptor cell population by a differentiation agent succinylated concanavalin A (Seigel and Notter, 1993). When a series of photoreceptor‐specific genes was examined for expression changes following UHRF1 depletion for a short term (4 days post‐lentiviral infection without further selection on puromycin) and long term (8 days' selection on puromycin in addition to the initial 4 days postinfection), all indicated genes were induced by the UHRF1 knockdown and the induction level was significantly higher in long‐term UHRF1‐knockdown cells than in short‐term knockdown cells (Fig. 4B,C). The higher gene induction along the duration of UHRF1 depletion suggested that these genes may get epigenetically derepressed through cell divisions. The derepression of photoreceptor‐related genes was also observed in Weri‐Rb1 cells upon stable (long‐term) UHRF1 knockdown although the gene induction was not as robust as in Y79 knockdown cells (Fig. S6). As HDAC can be recruited to specific loci for gene silencing by UHRF1 and other corepressor complexes (Formisano *et al.*, 2015; Unoki *et al.*, 2004), we tested whether HDAC inhibitors can further derepress the photoreceptor genes in combination with UHRF1 depletion. For this test, MS‐275 was chosen as it gave the highest and sustained accumulation of histone acetylation (Fig. 4D), and the MS‐275 treatment resulted in further induction of photoreceptor genes in UHRF1‐knockdown cells (Fig. 4E).

### 3.5 Increased histone H3 acetylation at photoreceptor gene promoters in UHRF1‐depleted retinoblastoma cells

**Figure 5:**
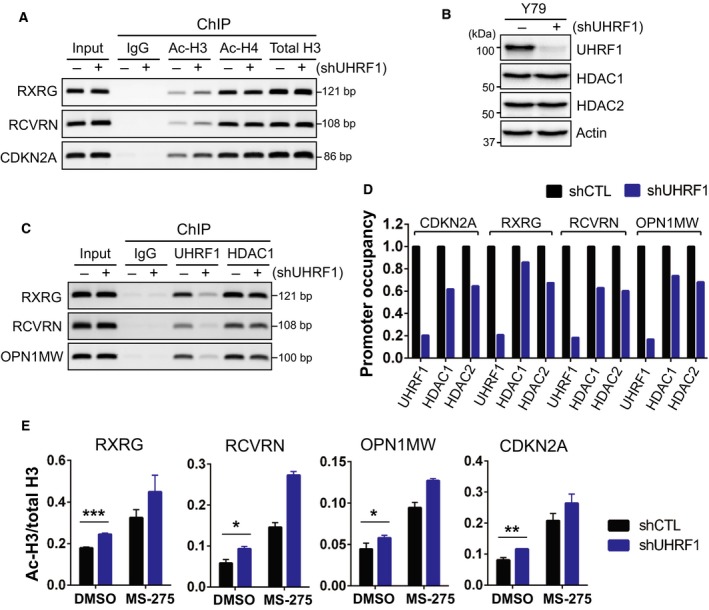
Increased histone H3 acetylation at photoreceptor gene promoters in UHRF1‐depleted RB cells. (A) ChIP‐PCR analysis for histone acetylation at indicated gene promoters in control (−) and UHRF1‐knockdown (+) Y79 cells. Promoter association of acetylated histone H3 (Ac‐H3) and H4 (Ac‐H4) is shown along with that of total histone H3. *CDKN2A* is a known UHRF1 target shown as a positive control for the analysis. (B) Immunoblots for indicated proteins in Y79 shCTL and shUHRF1 cells. (C) ChIP‐PCR analysis for UHRF1 and HDAC1 association at indicated gene promoters in shCTL and shUHRF1 Y79 cells. (D) Relative promoter occupancy of UHRF1 and HDACs at the indicated gene promoters determined by ChIP‐qPCR. The promoter association of each protein in shUHRF1 cells is shown, relative to that of shCTL cells. (E) ChIP‐qPCR analysis for histone H3 acetylation at indicated gene promoters in control and shUHRF1 Y79 cells treated with 0.5 µm MS‐275 for 2 days. The data are shown as the mean ± SD of normalized ratios of Ac‐H3/total H3 from three independent experiments. *P < 0.05, **P < 0.01, ***P < 0.001: unpaired Student's *t*‐test (two‐tailed).

To investigate whether the photoreceptor gene induction in UHRF1‐depleted RB cells is associated with increased histone acetylation at the gene promoters, we performed ChIP for histone acetylation marks on a subset of photoreceptor genes (Fig. 5A). As is the case with a known UHRF1 target *CDKN2A* (Unoki *et al.*, 2004), acetylation on histone H3 was higher for *RXRG* (retinoid X receptor γ) and *RCVRN* (recoverin) promoter in UHRF1‐knockdown cells than in control cells (Fig. 5A). The increase in histone H3 acetylation at the promoters was not due to changes in HDAC levels in UHRF1‐depleted cells (Fig. 5B). Consistent with the previous report that UHRF1 can recruit HDAC1 to promoters for gene repression (Unoki *et al.*, 2004), UHRF1 was found to be associated with several photoreceptor gene promoters, and HDAC binding to the identical promoter regions was modestly but consistently reduced in UHRF1‐depleted cells (Fig. 5C,D). The modest decrease in HDAC binding upon UHRF1 knockdown suggested that HDAC can be recruited to the promoters to a certain extent by residual UHRF1 and/or by other corepressor complexes in the cells. When we directly inhibited HDAC by MS‐275 treatment, higher histone H3 acetylation was observed for a subset of photoreceptor gene promoters (Fig. 5E), which correlated with increased gene expression by MS‐275 treatment (Fig. 4E). Of note, histone H4 acetylation at the photoreceptor gene promoters was not significantly different between UHRF1‐depleted cells and control cells (Fig. 5A and Fig. S7A), implying that acetylation status on histone H4 may not play a key role in transcriptional regulation. In addition, not all promoters of photoreceptor genes that are induced by UHRF1 depletion and MS‐275 treatment showed the accumulation of acetylated histone H3 in UHRF1‐depleted cells (Fig. S7B), suggesting that the expression of such genes may be regulated indirectly by other factors in the UHRF1‐knockdown cells. We reasoned that *RXRG* may be one of the factors as it is a critical transcription factor for photoreceptor development (Li *et al.*, 2003, 2002) and the *RXRG* expression is induced by UHRF1 depletion. When we examined the expression changes for a series of photoreceptor genes in *RXRG*‐knockdown Y79 cells, most of the examined genes were found to be downregulated (Fig. S8).

### 3.6 The growth‐inhibitory effects of MS‐275 in UHRF1‐depleted cells are mainly through inducing apoptosis

**Figure 6:**
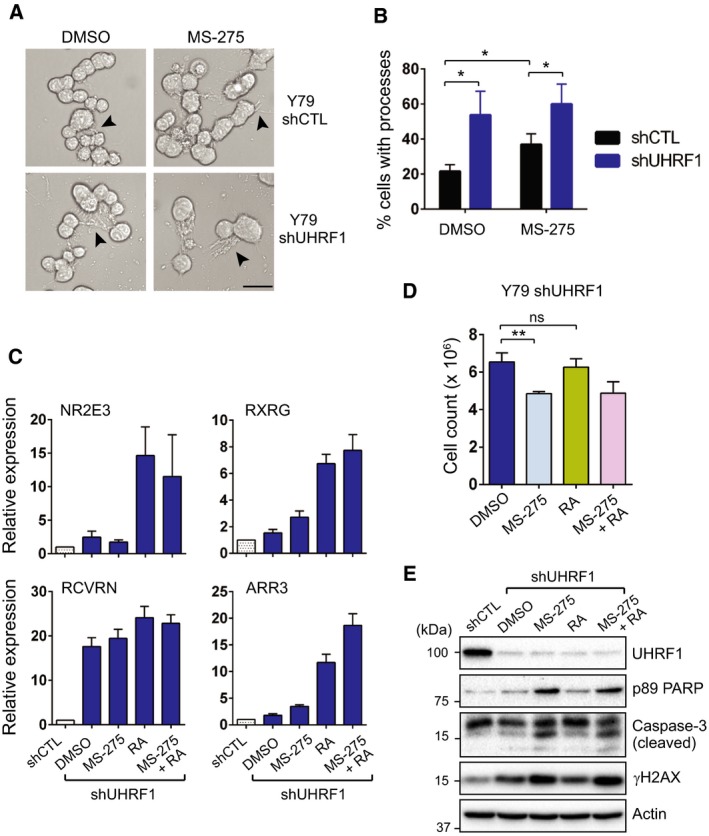
The growth‐inhibitory effects of MS‐275 in UHRF1‐depleted cells are mainly through inducing apoptosis. (A) Phase contrast images of control and UHRF1‐knockdown Y79 cells after culturing in serum‐free neurobasal differentiation medium with or without 1 µm MS‐275 for 24 h. Arrowheads mark the neurite‐like processes extended from the cells. Scale bar: 25 µm. (B) Quantification of the cells with processes. Over 600 total cells per group were examined for the morphological changes. The graph is shown as the mean ± SD from three independent experiments. *P < 0.05: unpaired Student's *t*‐test (two‐tailed). (C) Relative expression of photoreceptor genes in shUHRF1 Y79 cells subjected to a single or combined treatment with 0.5 µm MS‐275 and 1 µm RA for 48 h. The qRT‐PCR data are shown as the mean ± SD of fold changes from three independent experiments, relative to the normalized level in DMSO‐treated shCTL cells. (D) Live cell counts from the treatment groups of shUHRF1 cells shown in (C). **P < 0.01, ns: not significant; unpaired Student's *t*‐test (two‐tailed). (E) Immunoblots for indicated proteins in shUHRF1 Y79 cells treated with either a single agent or both agents as in (C), in comparison with DMSO‐treated shCTL Y79 cells.

As UHRF1 depletion derepresses the expression of photoreceptor genes in RB cells, we investigated whether UHRF1‐depleted cells would have a higher differentiation potential and whether this feature can contribute to growth inhibition in combination with MS‐275 treatment. When we examined the differentiation of UHRF1‐depleted cells by exposing to a neuronal differentiation medium with or without MS‐275, the UHRF1 knockdown alone caused significant morphological changes featured by outgrowth of neurite‐like processes (Fig. 6A). MS‐275 treatment only slightly increased the percentage of cells with morphological changes in UHRF1‐depleted cells while there was a significant increase in cells with processes in control‐knockdown cells upon MS‐275 treatment (Fig. 6B). Then, we investigated whether the photoreceptor gene induction and enhanced differentiation potential in UHRF1‐depleted cells can contribute to the increased growth‐inhibitory effects of MS‐275 that are observed in UHRF1‐knockdown cells (Fig. 1B). As the first step of the investigation, we determined the relative level of photoreceptor gene induction in comparison with RA, a well‐known differentiation agent which promotes neuron‐like morphological changes but does not induce significant apoptosis below 10 µm in RB cells (Conway *et al.*, 1997; Li *et al.*, 2003). As the control‐knockdown cells have photoreceptor gene repression mediated by intact UHRF1, we performed the comparisons only in UHRF1‐knockdown cells. The RA exerted much more potent effects on photoreceptor gene induction than MS‐275 in UHRF1‐depleted cells (Fig. 6C), however, did not cause any significant decrease in cell numbers in comparison with vehicle‐treated control whereas MS‐275‐treated cells showed a modest decrease in cell numbers during the same treatment time (Fig. 6D). These results suggested that the relatively lower photoreceptor gene induction in MS‐275‐treated UHRF1‐knockdown cells may not contribute to the decreased cell number as the RA‐treated counterparts with a higher differentiation potential based on the higher photoreceptor gene induction do not show any discernible growth‐inhibitory effects during the treatment time. When we further examined the basis for the modest decrease in cell counts of UHRF1‐depleted cells upon MS‐275 treatment, the growth‐inhibitory effect appeared to be caused by apoptosis although we used a lower dose of MS‐275 to reduce its apoptosis‐inducing effects and yet promote the derepression of photoreceptor genes for these assays (Fig. 6E). The lack of RA‐induced apoptosis may be one clue that differentiation‐associated changes might not contribute to apoptosis and more detailed understanding of differentiation effects driven by UHRF1 knockdown would be needed. However, as we have not observed any significant increase in G0/G1 population in HDAC inhibitor‐treated UHRF1‐knockdown cells (Fig. S2), the photoreceptor gene induction and morphological changes in the UHRF1‐depleted cells also appeared to be insufficient to promote terminal differentiation for growth inhibition. Taken together, these data suggest that the enhanced growth‐inhibitory effects of MS‐275 in UHRF1‐depleted cells are mainly through inducing apoptosis, rather than promoting terminal photoreceptor differentiation in cooperation with UHRF1 depletion.

### 3.7 UHRF1 depletion enhances therapeutic effects of HDAC inhibitor in orthotopic xenografts of retinoblastoma

**Figure 7:**
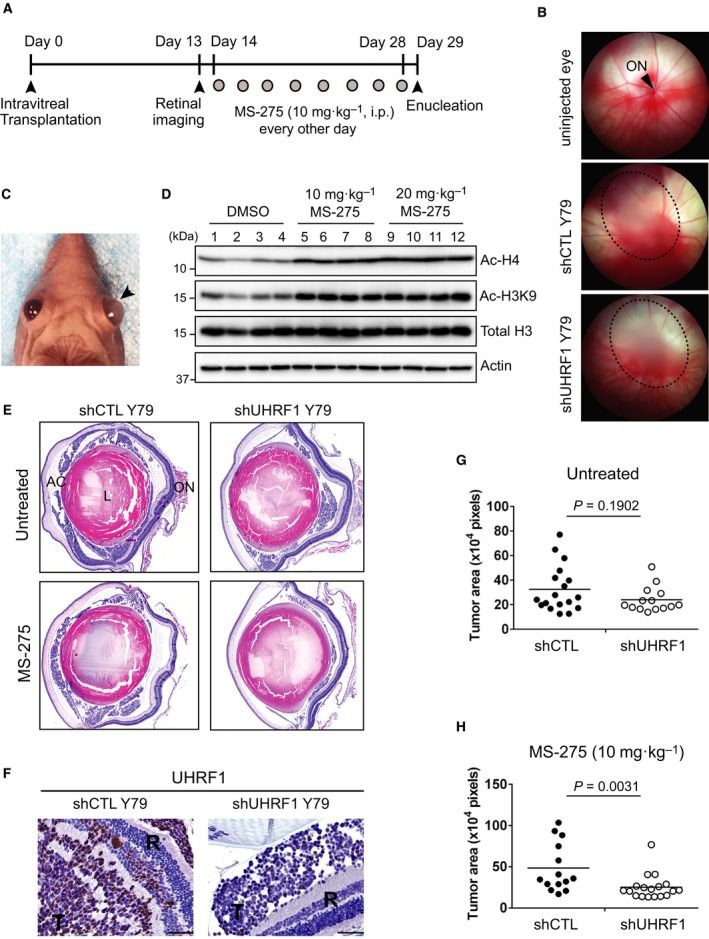
UHRF1 depletion enhances therapeutic effects of HDAC inhibitor in orthotopic xenografts of RB. (A) Schematic of orthotopic xenograft study. (B) Retinal imaging to monitor tumor development on day 13 post‐transplantation of shCTL and shUHRF1 Y79 cells. Xenografted eyes develop white cloud‐like tumors circled by dotted lines, whereas uninjected eyes show a clear retinal view. ON, optic nerve. (C) RB development in shCTL‐xenografted eye indicated by an arrowhead, in contrast to uninjected left eye. (D) Immunoblots for indicated proteins in retinal tissue lysates from the mice treated with either vehicle or two different doses of MS‐275 by intraperitoneal injection every other day for 2 weeks (four mice per group). (E) Representative images of tumor‐burdened eyes from each indicated group after H&E staining. AC, anterior chamber, L, lens, ON, optic nerve. (F) Immunostaining for UHRF1 on the indicated xenograft tumor sections. T: tumor, R: retina, scale bars: 50 µm. (G, H) Plots of average tumor area quantification in shCTL and shUHRF1 Y79 xenografts without treatments (G) or with MS‐275 treatments (H). Data points on the plots represent the average tumor area per section per mouse (*n* = 14–18 mice per group), and the horizontal bar of the dot plots indicates the mean value. The statistical analysis was performed by Mann–Whitney test (two‐tailed).

To determine the effects of UHRF1 depletion on the therapeutic efficacy of HDAC inhibitors *in vivo*, we established an orthotopic xenograft model of human RB by transplanting either control or UHRF1‐knockdown Y79 RB cells intravitreally into the eyes of immunocompromised mice (Fig. 7A). The xenografted mice were examined for tumor development by retinal imaging on day 13 post‐transplantation (Fig. 7B), and only the mice with detectable tumors were included in the study and subjected to MS‐275 treatment. Untreated xenografts of control cells typically develop conspicuous tumors featured by swollen eyeballs over 30–35 days post‐transplantation (Fig. 7C). Systemically administered MS‐275 can be delivered to the eyes of treated mice as evidenced by the increased histone acetylation in retinal tissues, and a low dose (10 mg·kg^−1^) of MS‐275 was adopted for the whole treatments as both high and low doses of MS‐275 resulted in a similar level of increase in histone acetylation in retina (Fig. 7D). Following the 2 weeks' treatment, tumor‐burdened eyes were analyzed for average tumor area to determine whether UHRF1 depletion would affect the therapeutic efficacy of MS‐275. Both control and UHRF1 knockdown cell‐injected eyes showed intravitreal tumor growth, which was often accompanied by tumor cell invasion into anterior chamber and retina (Fig. 7E). UHRF1 immunostaining verified that UHRF1 depletion in tumors derived from UHRF1‐knockdown Y79 cells was well maintained through the end of the study (Fig. 7F). Without MS‐275 treatment, UHRF1 depletion alone slightly reduced the mean value of tumor areas compared to the control cell‐injected eyes; however, the difference did not reach the statistical significance in our experimental conditions (Fig. 7G). When xenografted mice were treated with MS‐275, there was a significant reduction in tumor areas from UHRF1‐knockdown group in comparison with control‐knockdown counterparts (Fig. 7H). These data demonstrate that UHRF1 depletion may exert a marginal inhibitory effect on tumor growth but can clearly enhance the therapeutic efficacy of MS‐275 on tumor size reduction.

## 4 Discussion

Identification of novel genetic pathways or molecular targets whose disruption sensitizes cancer cells to preexisting chemotherapeutics would be beneficial to improve the efficacy of current chemotherapy. In line with this rationale, we recently discovered that UHRF1 downmodulation in RB cells increases the sensitivity to conventional genotoxic drugs, which are widely used for chemoreduction as the first‐line therapy for RB (He *et al.*, 2018). We found that UHRF1 depletion impedes DNA repair in RB cells by downregulating XRCC4 (X‐ray repair cross complementing 4) involved in nonhomologous end‐joining repair, which leads to higher apoptotic cell death in response to DNA damages induced by the genotoxic drugs.

In this study, we further evaluated the possibility of UHRF1 working as a target whose downmodulation can sensitize RB cells to HDAC inhibitors. The HDAC inhibitors are known to exert their growth‐inhibitory effects by several different mechanisms (Falkenberg and Johnstone, 2014). Apoptosis and cell cycle arrest can directly affect the cell viability and proliferation, and account for anticancer effects of HDAC inhibitors in many cancer cells. While cell cycle arrest was not obvious in UHRF1‐depleted RB cells upon HDAC inhibitor treatment, higher apoptosis was clearly observed in the UHRF1‐knockdown cells. Through further investigations for the mechanisms underlying the enhanced sensitivity to HDAC inhibitors in UHRF1‐depleted cells, we found that UHRF1 downmodulation significantly decreases the expression of GSTA4 and TXN2 in RB cells. Combined effects of each gene perturbation were sufficient to disturb the delicate balance for redox homeostasis in UHRF1‐depleted RB cells and augmented the ROS‐mediated apoptosis in response to HDAC inhibitors. Notably, GSTA4 is highly induced by HDAC inhibitor treatment in RB cells, indicative of its critical role in counteracting ROS accumulation driven by HDAC inhibitor treatment. Furthermore, the GSTA4 was reported to possess the highest glutathione peroxidase activity among the three mitochondrial GST isoforms purified from the mouse liver (Raza *et al.*, 2002). Considering that mitochondria are the major source of intracellular ROS (Zorov *et al.*, 2014) and the mitochondrial thioredoxin TXN2 is significantly reduced in UHRF1‐depleted cells, it is plausible that even modest perturbation in GSTA4 expression may exert substantial effects on ROS detoxification in response to HDAC inhibitors. Indeed, depletion of GSTA4 alone was found to induce massive DNA damage and apoptosis in RB cells and further sensitized the cells to HDAC inhibitors. These results suggest a possibility that selective modulation of mitochondrial GST isoenzymes in UHRF1‐depleted RB cells may determine the susceptibility to ROS‐mediated apoptosis. Currently, how UHRF1 regulates the expression of GSTA4 and TXN2 remains unclear; however, the transcriptional regulation is likely to be indirect effects mediated by other UHRF1 targets since UHRF1 works mostly as a transcriptional repressor by recruiting repressive chromatin modifiers onto the target promoters (Kim *et al.*, 2009; Unoki *et al.*, 2004). As UHRF1 downmodulation can sensitize RB cells to both standard genotoxic drugs and HDAC inhibitors, combination therapy using these two types of drugs is expected to be efficacious, while doses of each drug can be reduced to achieve similar or better therapeutic effects with decreased cytotoxicity for noncancer cells. Alternatively, UHRF1 targeting may allow for the rational choice of either type of drugs in the course of RB treatment as the identified mechanisms underlying the potential therapeutic effects of UHRF1 depletion such as defects in DNA repair and intracellular ROS detoxification may affect the efficacy of both types of drugs. As UHRF1 is not expressed in normal retina (Kan *et al.*, 2017), local UHRF1 targeting in the eyes is expected to result in selective therapeutic effects with these drugs.

**Figure 8:**
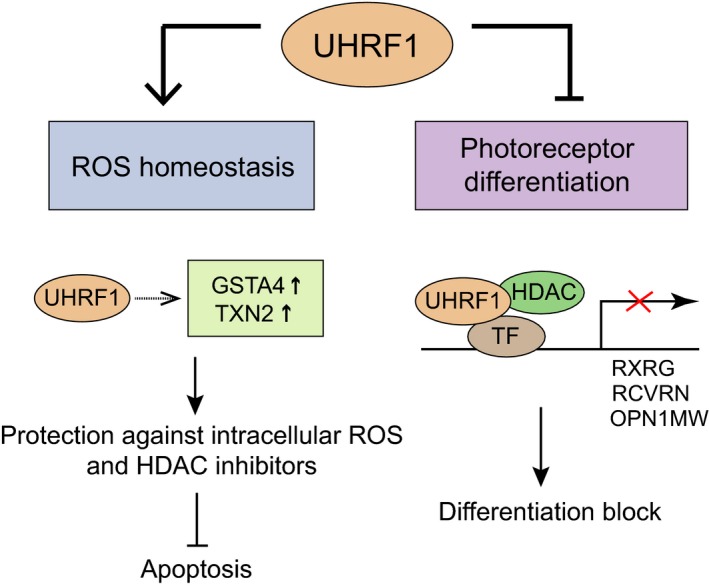
Proposed functions of UHRF1 in RB cells identified in this study. UHRF1 regulates ROS‐responsive genes to counteract the accumulation of intracellular ROS that RB cells may encounter due to their high metabolic activities for robust proliferation. This new function of UHRF1 may also contribute to protection against ROS‐generating drugs such as HDAC inhibitors to evade apoptotic cell death. On the other hand, UHRF1 participates in repression of photoreceptor differentiation as a corepressor in a multiprotein complex containing HDAC and presumably transcription factor (TF) to repress the photoreceptor‐specific genes.

Another potential mechanism for HDAC inhibitors to exert growth‐inhibitory effects in cancer cells is induction of cellular differentiation through lineage‐specific gene expression and cell cycle exit (Ceccacci and Minucci, 2016; Falkenberg and Johnstone, 2014). In fact, differentiation therapy has been a promising therapeutic strategy for the treatment of acute myeloid leukemia by inducing myeloid differentiation with epigenetic drugs (Bots *et al.*, 2014; Saunthararajah *et al.*, 2012). We discovered that UHRF1 directly represses the expression of photoreceptor genes in Y79 cells in cooperation with HDACs and also indirectly by its downstream target. This finding led us to a possibility that UHRF1 depletion in combination with treatment of HDAC inhibitors or other differentiation agents may exert significant growth‐inhibitory effects in Y79 cells by promoting photoreceptor‐like differentiation. Although MS‐275 treatment further upregulated photoreceptor gene expression in UHRF1‐depleted Y79 cells and increased the frequency of morphological changes, the growth‐inhibitory effects of MS‐275 were mainly through inducing apoptosis rather than differentiation‐mediated growth arrest. In our experimental conditions, prolonged treatment with HDAC inhibitors beyond 2–3 days induced a substantial level of apoptosis in UHRF1‐depleted cells even at low concentrations (data not shown), rendering the contribution of cell differentiation to the growth‐inhibitory effects very marginal if any. An early study reported that Y79 RB cells pretreated with retinoic acid/NaBu *in vitro* did not develop tumors after subretinal transplantation of the treated cells into immunosuppressed rats, implying that tumorigenicity of Y79 cells can be suppressed by drug‐induced *in vitro* differentiation (del Cerro *et al.*, 1992). However, another study reported that massive morphological differentiation of Y79 cells by neurotrophic agents was not sufficient for suppressing tumor formation upon subretinal transplantation (Seigel *et al.*, 1994). These results suggest that extensive morphological differentiation *in vitro* may not indicate that the cells are mitotically arrested to suppress tumorigenicity although specific differentiation markers are expressed to cause the neuron‐like morphological changes. This appears to be the case for our experimental settings as UHRF1‐depleted Y79 cells treated with retinoic acid express much higher levels of photoreceptor genes than those treated with MS‐275 but do not show any decrease in cell proliferation based on the live cell counts. Nevertheless, it is worth noting that UHRF1 participates in repression of photoreceptor differentiation in RB cells at least in part. As poorly differentiated RB is associated with multiple high‐risk histopathologic factors to some extent (Kashyap *et al.*, 2012), the negative role of UHRF1 in photoreceptor differentiation may present a novel insight into the tumor‐promoting functions of UHRF1 in RB cells in addition to its implication in ROS homeostasis and protective roles against HDAC inhibitor‐induced cell death (Fig. 8).

## 5 Conclusions

In summary, we presented experimental evidences documenting that UHRF1 downmodulation can sensitize RB cells to HDAC inhibitors by augmenting oxidative stress‐mediated apoptosis via downregulation of GSTA4 and TXN2, along with a newly identified role for UHRF1 in epigenetic repression of photoreceptor genes in RB cells. Therefore, this study provides further mechanistic insights into how UHRF1 targeting may be beneficial for combination therapies with other drugs to improve the efficacy of current chemotherapy for RB.

## Conflict of interest

The authors declare no conflict of interest.

## Author contributions

JKK designed the study and wrote the manuscript. JKK, GK, YM, ZW, XT, and HH performed the experiments and analyzed the data. CL contributed to the RNA‐sequencing data analysis.

## Supporting information


**Fig. S1.** Increased apoptosis in response to HDAC inhibitors upon short‐term UHRF1 knockdown or different shRNA clone‐mediated UHRF1 knockdown.
**Fig. S2.** Treatment with HDAC inhibitors does not alter cell cycle profiles in control and UHRF1‐knockdown Y79 cells.
**Fig. S3.** Expression of GSTA4 and TXN2 in response to HDAC inhibitors upon short‐term UHRF1 knockdown or different shRNA clone‐mediated UHRF1 knockdown.
**Fig. S4.** p38 phosphorylation and NRF2 expression in shUHRF1 Y79 cells in response to HDAC inhibitors.
**Fig. S5.** Gene ontology (GO) analysis of upregulated genes in stable shUHRF1 Y79 cells.
**Fig. S6.** Stable UHRF1 knockdown in Weri‐Rb1 cells upregulates expression of a subset of photoreceptor‐related genes.
**Fig. S7.** ChIP analysis for histone acetylation at photoreceptor gene promoters.
**Fig. S8.** *RXRG* knockdown in Y79 cells downregulates expression of photoreceptor‐related genes.
**Table S1.** Primers used in this study.

**Table S2.** List of differentially expressed genes in shUHRF1 Y79 cells.

## Data Availability

The RNA‐seq data in this study were deposited in the NCBI Gene Expression Omnibus (GEO) database under the accession number http://www.ncbi.nlm.nih.gov/geo/query/acc.cgi?acc=GSE135424.
