# Supplementary material for: UHRF1 downmodulation enhances antitumor effects of histone deacetylase inhibitors in retinoblastoma by augmenting oxidative stress‐mediated apoptosis
Source: Mol Oncol. 2019 Dec 13;14(2):329–46. doi: 10.1002/1878-0261.12607 (PMC6998393; doi:10.1002/1878-0261.12607)
Supplement: Supplementary file 1 — Fig. S1. Increased apoptosis in response to HDAC inhibitors upon short‐term UHRF1 knockdown or different shRNA clone‐mediated UHRF1 knockdown. Fig. S2. Treatment with HDAC inhibitors does not alter cell cycle profiles in control and UHRF1‐knockdown Y79 cells. Fig. S3. Expression of GSTA4 and TXN2 in response to HDAC inhibitors upon short‐term UHRF1 knockdown or different shRNA clone‐mediated UHRF1 knockdown. Fig. S4. p38 phosphorylation and NRF2 expression in shUHRF1 Y79 cells in response to HDAC inhibitors. Fig. S5. Gene ontology (GO) analysis of upregulated genes in stable shUHRF1 Y79 cells. Fig. S6. Stable UHRF1 knockdown in Weri‐Rb1 cells upregulates expression of a subset of photoreceptor‐related genes. Fig. S7. ChIP analysis for histone acetylation at photoreceptor gene promoters. Fig. S8. RXRG knockdown in Y79 cells downregulates expression of photoreceptor‐related genes. Table S1. Primers used in this study. [file MOL2-14-329-s001.pdf]

Supporting information

Supplementary Table S1. Primers used in this study

**Quantitative RT-PCR**	
(1) TXNIP	Product size
Forward: 5'-GGCTAAAGTGCTTTGGATGCA-3'	
Reverse: 5'-TCTCATGATCACCATCTCATTCTCA-3'	124 bp
(2) GSTA4	
Forward: 5'-GTAATCAGCTGAGCCTTGCAG-3'	
Reverse: 5'-GCTGCCAGGTTCAAGGAATCT-3'	158 bp
(3) TXN	
Forward: 5'-GTGAAGCAGATCGAGAGCAAG-3'	
Reverse: 5'-CGTGGCTGAGAAGTCAACTACTA-3'	87 bp
(4) TXN2	
Forward: 5'-CTGGTGGCCTGACTGTAACAC-3'	
Reverse: 5'-TGACCACTCGGTCTTGAAAGT-3'	111 bp
(5) NOXO1	
Forward: 5'-AGATCAAGAGGCTCCAAACG-3'	
Reverse: 5'-GGAAGGTCTCCTTGAGGGTCT-3'	120 bp
(6) Actin	
Forward: 5'-AGAGCTACGAGCTGCCTGAC-3'	
Reverse: 5'-AGCACTGTGTTGGCGTAC-3'	184 bp
(7) NR2E3	
Forward: 5'-GAAACCTGTGCTAAGCTGGAGC-3'	
Reverse: 5'-GAGGTCTCATGGATGCTGTCCA-3'	131 bp
(8) RXRG	
Forward: 5'-CAGAAGTGCCTTGTCATGG-3'	
Reverse: 5'-CCTCACTCTCAGCTCGCTCT-3'	82 bp
(9) RCVRN	
Forward: 5'-CCAGAGCATCTACGCCAAGT-3'	
Reverse: 5'-CACGTCGTAGAGGGAGAAGG-3'	187 bp
(10) GNAT1	
Forward: 5'-GAGGACGCTGAGAAGGATGC-3'	
Reverse: 5'-TGGATGTTGAGTGTGGTCAT-3'	209 bp
(11) GNAT2	
Forward: 5'-GAGCCAGTGCTGAGGACAAA-3'	
Reverse: 5'-CCAGGCATTCTTCTGGTGAA-3'	192 bp
(12) ARR3	
Forward: 5'-GCACAATTATTAGACCGGG-3'	
Reverse: 5'-GCCTTTCCGCGTAAAC-3'	218 bp
(13) OPN1MW	
Forward: 5'-TCACCCCACTCAGCATCATC-3'	
Reverse: 5'-GAAGCAGAATGCCAGGACCAT-3'	152 bp
(14) OPN1LW	
Forward: 5'-CATCATCCCACTCGCTATCATCAT-3'	
Reverse: 5'-GACGCAGTACGCAAAGATCATC-3'	154 bp

(15) NRF2	
Forward: 5'-CACATCCAGTCAGAAACCAGTGG-3'	
Reverse: 5'-GGAATGTCTGCGCCAAAAGCTG-3'	112 bp
**ChIP-PCR**	
(1) RXRG	
Forward: 5'-GGTGGCCGGAAGCATTTATC-3'	
Reverse: 5'-ACCTCCACATCAAGCCAACC-3'	121 bp
(2) RCVRN	
Forward: 5'-GTCCATCAGTCACAGGGAGT-3'	
Reverse: 5'-CCCATGTGACCACGTTCTCT-3'	108 bp
(3) ARR3	
Forward: 5'-CCATGAGCGGTCATACGAGG-3'	
Reverse: 5'-ATCTGTGTCACCCCTACCGA-3'	121 bp
(4) OPN1MW	
Forward: 5'-AGCAAGGAAGCAAGGGGTG-3'	
Reverse:5'-CGGGCCTCTTCACCTTAAAA-3'	100 bp
(5) OPN1LW	
Forward: 5'-AGCTGATGGAAGCCGTGAAA-3'	
Reverse: 5'-CTGTTAGTGCCCAACTCCGT-3'	122 bp
(6) CDKN2A	
Forward: 5'-GGGCTCTCACAACTAGGAAAG-3'	
Reverse: 5'-GGGTGTTTGGTGTCATAGGG-3'	86 bp

Supplementary Table S2. List of differentially expressed genes (shUHRF1 vs. shCTL)

Due to the space limit, Supplementary Table S2 is provided in a separate file.

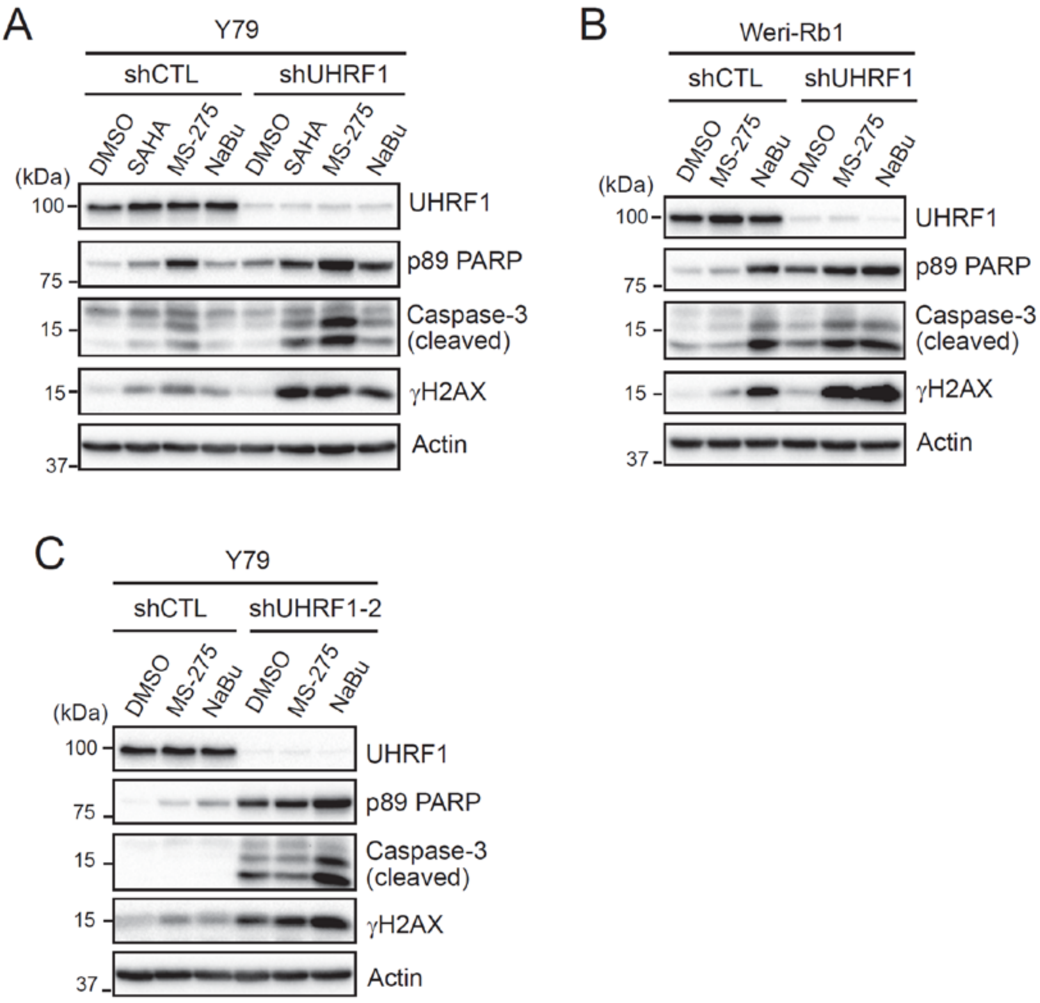

Supplementary Fig. S1. Increased apoptosis in response to HDAC inhibitors upon short-term UHRF1 knockdown or different shRNA clone-mediated UHRF1 knockdown. (A) Immunoblots for indicated proteins upon short-term UHRF1 knockdown (4 days post-infection) in Y79 cells using the shUHRF1-1 clone. The knockdown cells were treated with 1 µM SAHA, 1 µM MS-275, and 1 mM NaBu for 48 h. (B) Expression of indicated proteins in Weri-Rb1 short-term UHRF1-knockdown cells (4 days post-infection) using the shUHRF1-1 clone. After the acute knockdown, cells were treated with 1 µM MS-275 or 1 mM NaBu for 20 h. (C) Expression of indicated proteins in Y79 stable UHRF1-knockdown cells (7 days' selection on puromycin in addition to the initial 4 days post-infection) using the shUHRF1-2 clone. Cells were treated as in (A) for 24 h.

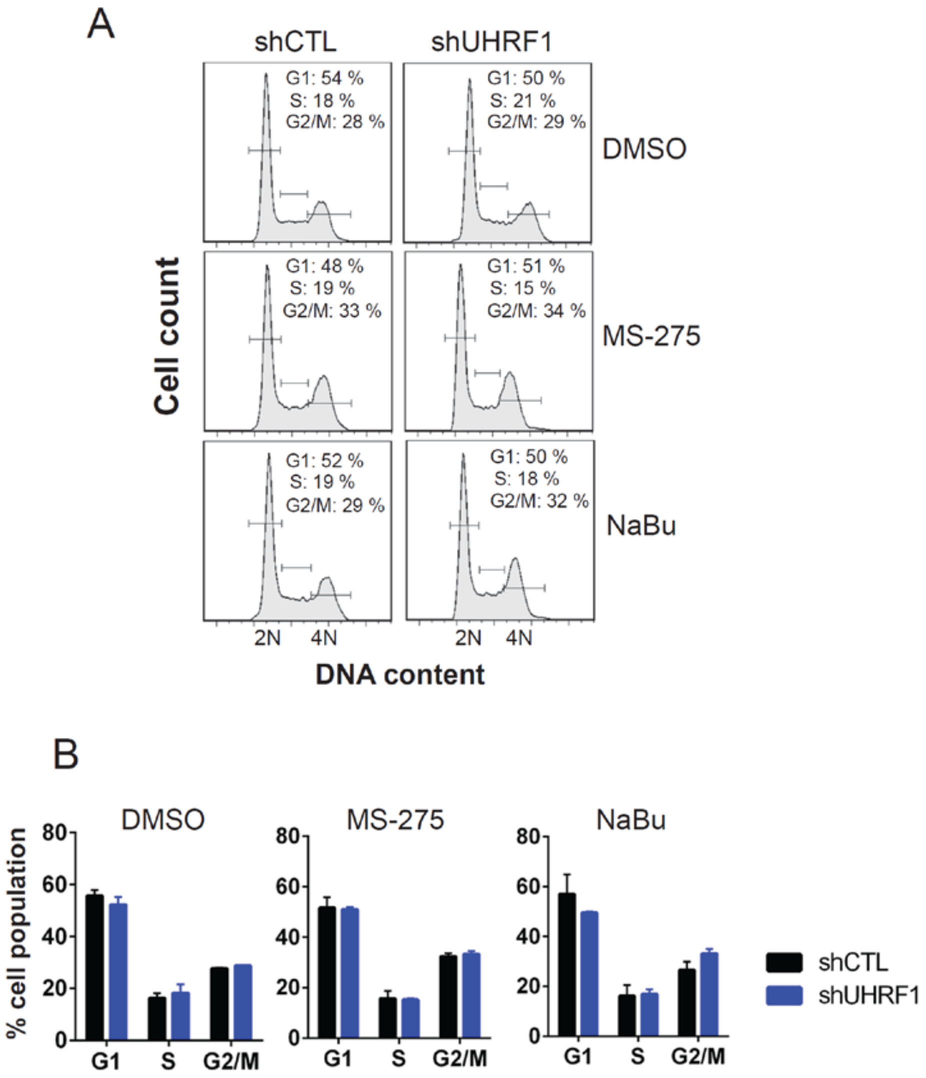

Supplementary Fig. S2. Treatment with HDAC inhibitors does not alter cell cycle profiles in control and UHRF1-knockdown Y79 cells. (A) Representative cell cycle profiles of shCTL and shUHRF1 Y79 cells treated with 1 µM MS-275 or 1 mM NaBu for 24 h. (B) Graphs showing the % cell population at each cell cycle phase in Y79 shCTL and shUHRF1 cells treated with indicated HDAC inhibitors as in (A). The graphs are shown as the mean ± SD from two independent experiments.

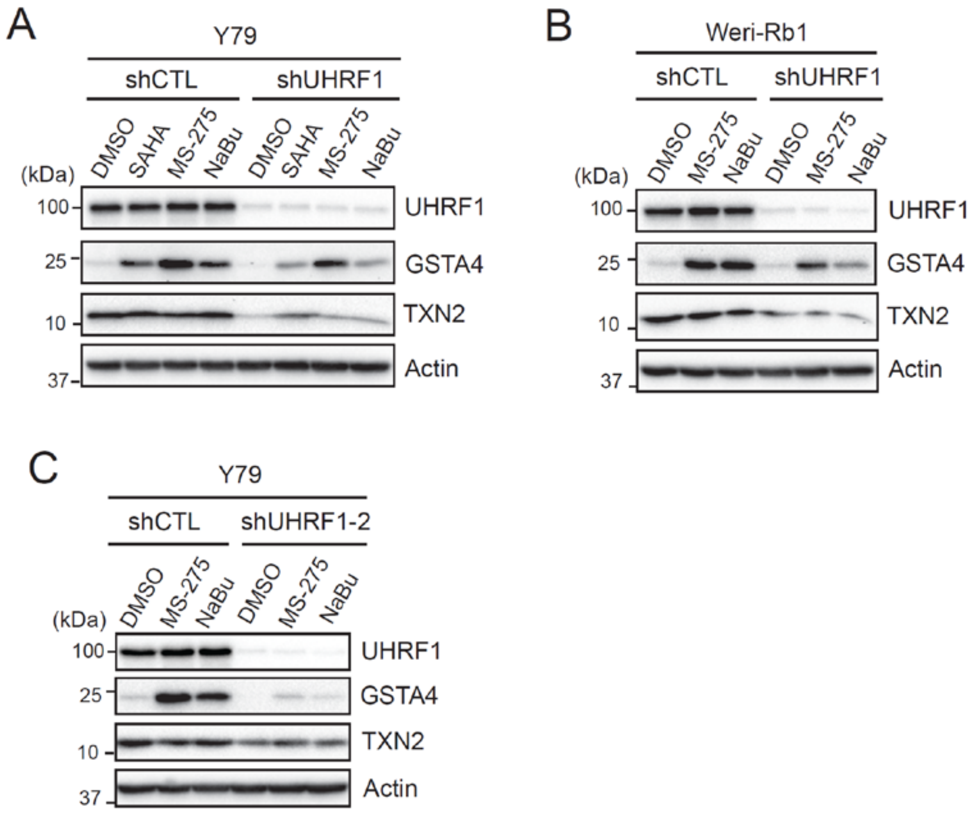

Supplementary Fig. S3. Expression of GSTA4 and TXN2 in response to HDAC inhibitors upon short-term UHRF1 knockdown or different shRNA clone-mediated UHRF1 knockdown. (A) Immunoblots for indicated proteins upon short-term UHRF1 knockdown (4 days post-infection) in Y79 cells using the shUHRF1-1 clone. The knockdown cells were treated with 1 µM SAHA, 1 µM MS-275, and 1 mM NaBu for 48 h. (B) Expression of indicated proteins in Weri-Rb1 short-term UHRF1-knockdown cells (4 days post-infection) using the shUHRF1-1 clone. After the acute knockdown, cells were treated with 1 µM MS-275 or 1 mM NaBu for 20 h. (C) Expression of indicated proteins in Y79 stable UHRF1-knockdown cells (7 days' selection on puromycin in addition to the initial 4 days post-infection) using the shUHRF1-2 clone. Cells were treated as in (A) for 24 h.

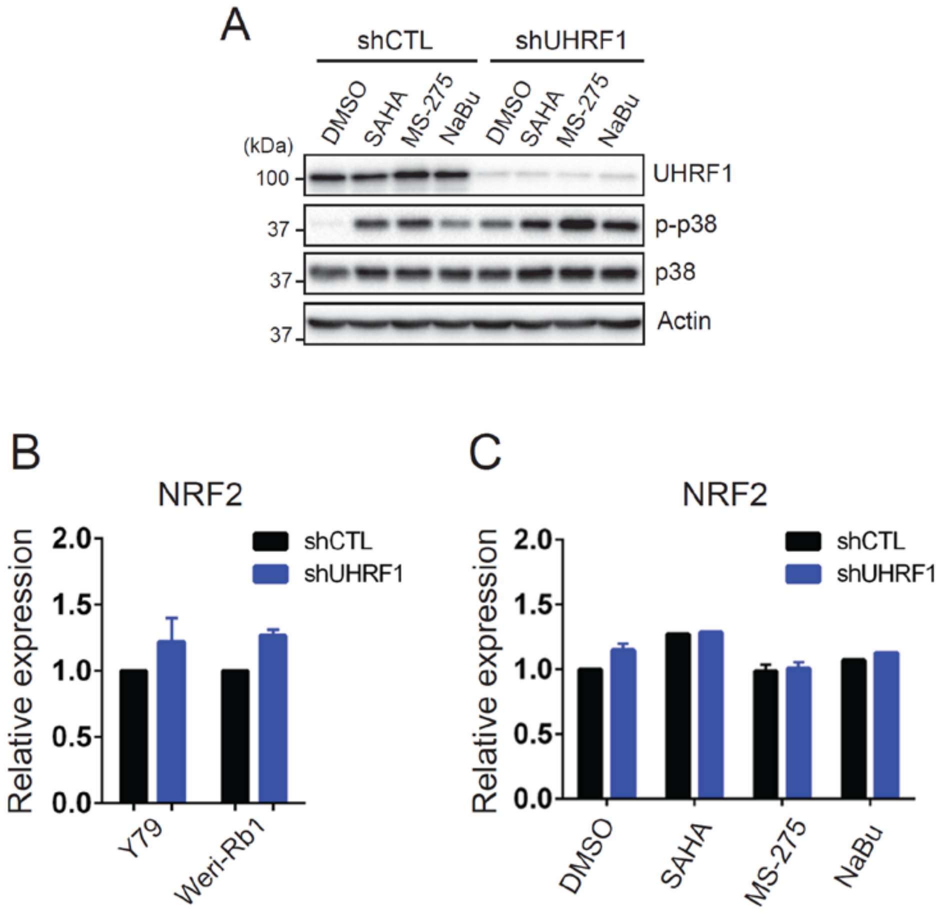

Supplementary Fig. S4. p38 phosphorylation and NRF2 expression in shUHRF1 Y79 cells in response to HDAC inhibitors. (A) Immunoblots for indicated proteins in Y79 shCTL and shUHRF1 cells after treatment with 1 µM SAHA, 1 µM MS-275, and 1 mM NaBu for 48 h. (B) qPCR analysis for basal expression changes of NRF2 after UHRF1 knockdown in Y79 and Weri-Rb1 cells. The data are shown as the mean ± SD of fold changes from three independent experiments, relative to the normalized level in each control-knockdown group. (C) Expression of NRF2 in Y79 shUHRF1 cells in response to HDAC inhibitors, determined by qPCR analysis. Cells were treated with 1 µM SAHA, 1 µM MS-275, and 1 mM NaBu for 24 h. The results are shown as the mean ± SD of fold changes from two independent experiments, relative to the normalized level in DMSO-treated shCTL cells.

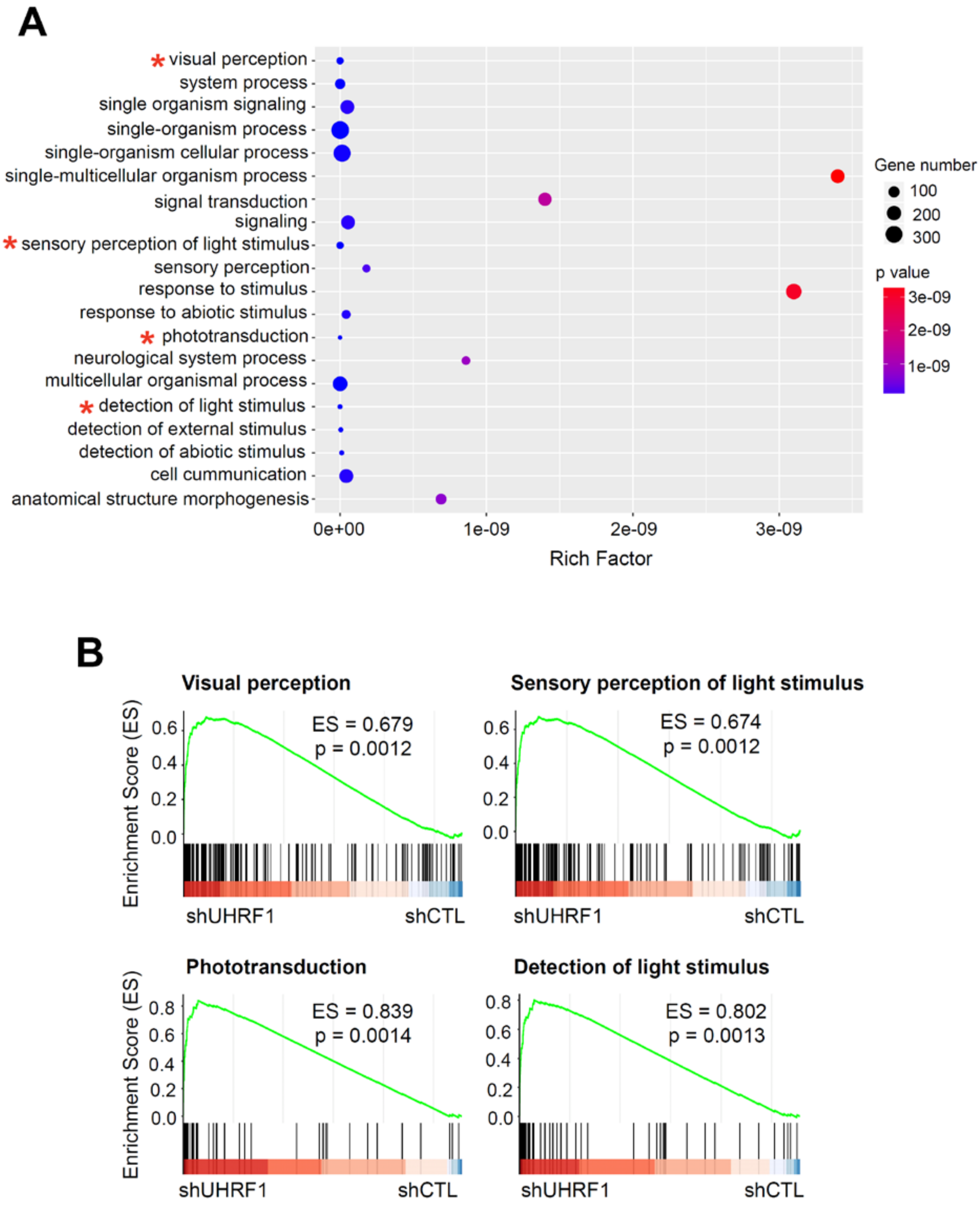

Supplementary Fig. S5. Gene ontology (GO) analysis of upregulated genes in stable UHRF1-knockdown Y79 cells. (A) Plot showing the statistics of GO enrichment for the top 20 enriched GO terms for biological process aspect. The top 20 enriched GO terms are ranked by p-values which are shown as Rich Factor in X-axis. The size of dots on the plot indicates the gene number belonging to that specific GO term. The GO terms related to photoreceptor and phototransduction are marked with a red asterisk. (B) GSEA (gene set enrichment analysis) plots with the enrichment score (EC) and p-value for the indicated GO gene sets.

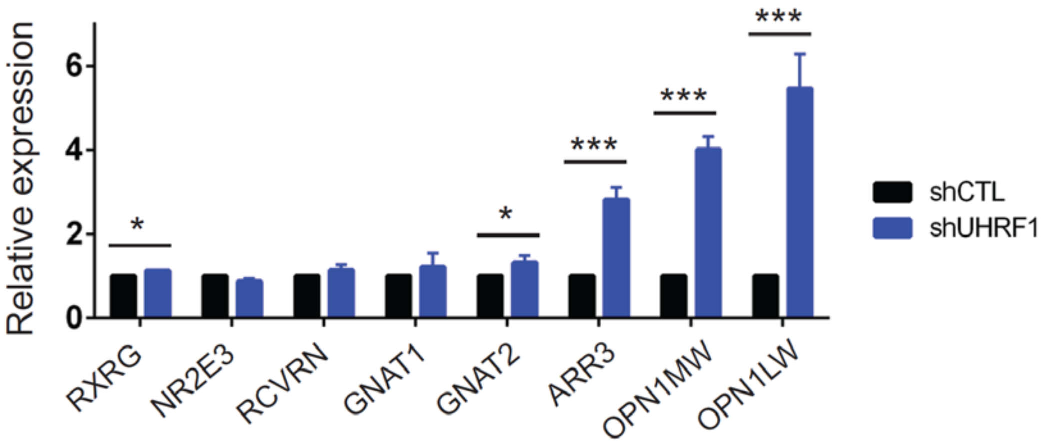

Supplementary Fig. S6. Stable UHRF1 knockdown in Weri-Rb1 cells upregulates expression of a subset of photoreceptor-related genes. The qRT-PCR data are shown as the mean ± SD of fold changes from three independent experiments, relative to the normalized level in each control-knockdown group. * $P < 0.05$, *** $P < 0.001$: unpaired student's t-test (two-tailed).

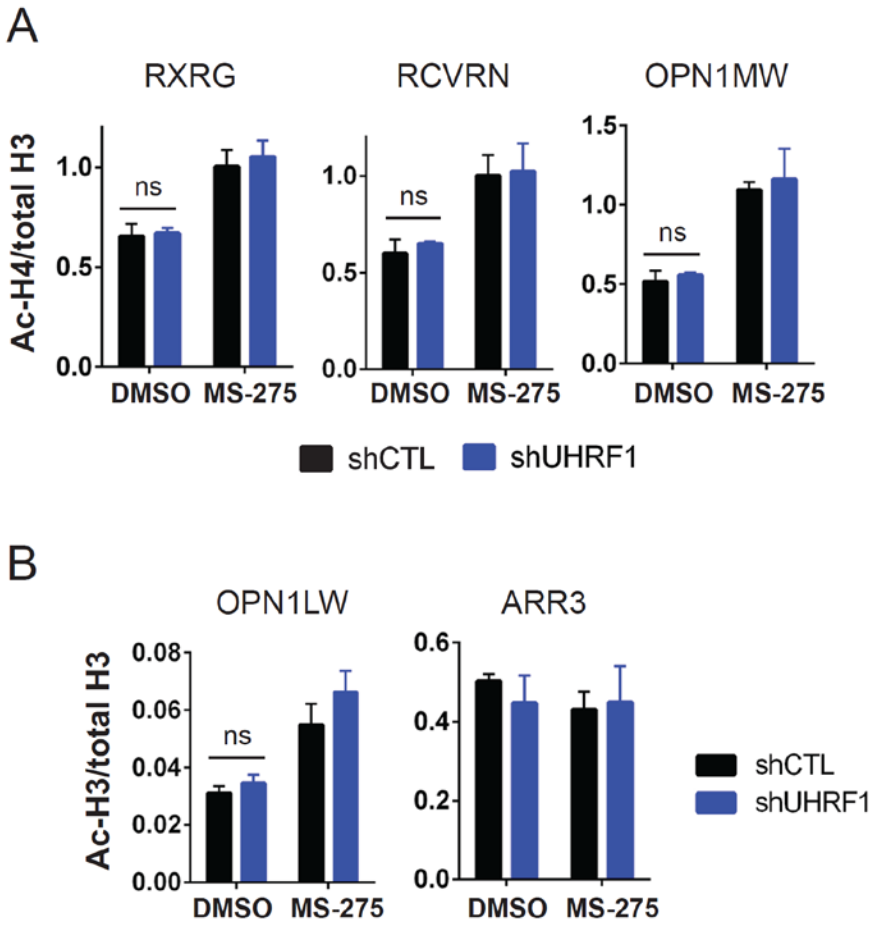

Supplementary Fig. S7. ChIP analysis for histone acetylation at photoreceptor gene promoters. (A) ChIP-qPCR analysis for histone H4 acetylation at the indicated gene promoters in control and shUHRF1 Y79 cells treated with 0.5 µM MS-275 for 2 days, demonstrating that histone H4 acetylation at photoreceptor gene promoters does not change upon UHRF1 depletion for both vehicle control and MS-275 treatment groups. The data are shown as the mean ± SD of normalized ratios of Ac-H4/total H3 from three independent experiments. ns: not significant; unpaired student's t-test (two-tailed). (B) ChIP-qPCR analysis for histone H3 acetylation, showing that not all photoreceptor gene promoters exhibit increased histone H3 acetylation upon UHRF1 knockdown in Y79 cells. The normalized ratios of Ac-H3/total H3 are shown for the indicated gene promoters in cells treated as in (A).

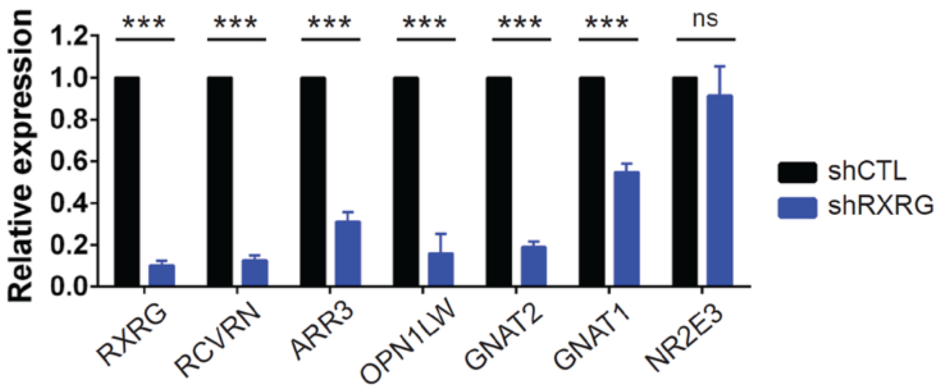

Supplementary Fig. S8. *RXRG* knockdown in Y79 cells downregulates expression of photoreceptor-related genes. The qRT-PCR data are shown as the mean ± SD of fold changes from four biological replicates, relative to the normalized level in each control-knockdown group. *** $P < 0.001$, ns: not significant; unpaired student's t-test (two-tailed).
